# Beyond social cues: the normative influence of social referents on COVID-19 vaccine compliance among Latino day laborers

**DOI:** 10.1007/s10865-026-00709-8

**Published:** 2026-09-16

**Authors:** Nanjun Chen, Karen Del Rio, Vahed Maroufy, Maria Eugenia Fernández-Esquer

**Affiliations:** School of Public Health, The University of Texas Health Science Center at Houston, Houston, TX USA

**Keywords:** Latino day laborers, Descriptive norms, Injunctive norms, Normative beliefs, COVID-19 vaccination

## Abstract

Pandemic uncertainty led Latino day laborers (jornaleros) to rely on their close networks for direction. Social-norms research distinguishes descriptive norms (what trusted others do), injunctive norms (what they say one should do), and normative beliefs (how much they care about compliance); yet the relative weight of family, friends, coworkers, and neighbors in shaping vaccine choices remains unknown. To compare the influence of descriptive and injunctive norms from each referent group on COVID-19 vaccination among Latino day laborers in Houston, Texas. Two cross-sectional surveys (Phase 1 N = 199; Phase 2 N = 359) were conducted at 14 hiring corners in 2021–2023. Three distinct multivariable logistic regression models were constructed to assess the associations between vaccination compliance status and (1) individual norm items, (2) overall norm scores across referents, and (3) referent-specific norm scores, adjusting for demographic characteristics. In Phase 1, family descriptive norms were significantly associated with vaccination compliance (aOR = 1.976, 95% CI [1.297–3.011], *p* = 0.002). Additionally, the cumulative descriptive norm score across referents was significantly associated with vaccine compliance (aOR = 2.443, 95% CI [1.213, 4.918], *p* = 0.012). In Phase 2, the cumulative normative beliefs score across referents was significantly associated with vaccination compliance (aOR = 1.347, 95% CI [1.004, 1.807], *p* = 0.047). A subsequent referent-specific analysis confirmed this effect was driven exclusively by family norms (aOR = 1.677, 95% CI [1.141, 2.466], *p* = 0.009). In the absence of clear institutional guidance, family remained the decisive normative referent for jornaleros, surpassing physically co-present referents such as coworkers and fellow day laborers, even when family members were likely geographically distant or located outside the United States. Interventions must leverage these kin-based norms, and sustained funding is critical to expand culturally grounded research and strengthen the Latino public-health workforce. Identify how distinct social referent groups influence vaccination behavior among Latino day laborers and assess how family-centered norms can inform and potentially transform future public health interventions.

**Supplementary Information:**

The online version contains supplementary material available at https://doi.org/10.1007/s10865-026-00709-8.

## Introduction

The COVID-19 pandemic has highlighted the pervasive yet frequently overlooked influence of social norms on pandemic-related health behaviors. As a social process understood to shape both prosocial and antisocial actions in diverse contexts (Schultz, 2022), the influence of social norms may have been critical particularly during the early stages of the pandemic, a time when uncertainty about the nature of the virus, its transmission, and appropriate preventive measures left individuals reliant on the influence of others and perceived group norms to guide behavior (Bavel et al., 2020; Zhou et al., 2023). During this early period, scientific knowledge about COVID-19 was evolving, and official guidance was inconsistent, leading people to look to social cues to guide their preventive actions. This reliance on others may have been crucial because, under uncertain conditions, observing others' behavior provides practical guidance on how to behave appropriately (Cialdini et al., 1991; Lapinski & Rimal, 2005). For instance, Van Bavel et al. (2020) demonstrated that simply reminding people that most of their community was already adhering to COVID preventive practices increased reported compliance, underscoring the power of normative cues when formal guidance from public health sources may have been perceived as unclear.

## Social norms and referents

While social norms encompass different dimensions of rules and expectations that guide compliant behavior, three constructs are critical for understanding their role during the COVID pandemic: descriptive norms, injunctive norms, and normative beliefs. Descriptive norms capture perceptions of what most people do, the perceived prevalence of a behavior, for example, noticing that most customers wear masks in a store (Cialdini et al., 1991; Lapinski & Rimal, 2005). Injunctive norms reflect perceptions of expected behaviors that others approve or disapprove of, such as whether one ought to wear a mask to protect the community (Foust & Taber, 2024; White et al., 2009). Normative beliefs, drawn from the Theory of Planned Behavior (Ajzen, 1991), capture how much specific referents are perceived to care about or be invested in an individual’s behavior (Lapinski & Rimal, 2005). Although related to injunctive norms, the two constructs differ in emphasis: injunctive norms codify beliefs about right and wrong, and center on perceived expectations or approval (what important others think one should do), whereas normative beliefs, as operationalized in the present study, reflect the perceived degree of concern or investment that referent groups have in the individual’s behavior. Importantly, this study does not test the full TPB normative pathway, as motivation-to-comply items and behavioral intentions were not separately measured. Together, these concepts influence health behavior in complementary ways. For example, descriptive norms signal what is common, while injunctive norms indicate what is acceptable, and normative beliefs represent fundamental assumptions that may influence whether individuals feel compelled to act on those signals.

The relative strength of these normative influences depends on the salience of the norm, whether the individual is aware of it and perceives it as relevant to a particular situation (Cialdini et al., 1991). Lapinski and Rimal (2005) further distinguish between collective norms, i.e., the actual distribution of behaviors or attitudes within a group, and perceived norms (individual interpretations of those behaviors). Perceived norms are often the more powerful driver of behavior, as people tend to respond to what they believe others expect of them rather than to objective behavioral trends or standards (Lapinski & Rimal, 2005). During the pandemic, these social influence dynamics were embedded within processes of social identity, as individuals interpreted descriptive and injunctive cues through the lens of groups they perceived as meaningful. For example, for Latino day laborers, critical referents may include different constituents of their social network, such as family members, friends, coworkers, and neighbors (Negi et al., 2013). As such, Latino day laborers may have aligned their preventive actions with the perceived behavioral norms of groups they identified with or aspired to belong to, even when those norms may have diverged from official public health recommendations (Zhou et al., 2023). Thus, the influence of descriptive norms, injunctive norms, and normative beliefs depends on the referent group and the extent to which the individual identifies with it.

The present study applies this theoretical framework to Latino day laborers (jornaleros), a population for whom social norms may carry particular weight in shaping health decisions. Latino day laborers are predominantly foreign-born men employed in informal, cash-based labor markets, frequently without access to reliable institutional health information, consistent public health outreach in their preferred language, or formal employment protections. These conditions heighten dependence on close social networks for behavioral cues, making this population a theoretically important test case for understanding normative influence under conditions of compounded uncertainty. Importantly, the social settings in which day laborers enact health practices may not fully overlap with the social ties that shape their perceived norms: workers gather at hiring corners with peers and coworkers, while also maintaining family and social obligations that may extend across countries of origin (Fernández-Esquer et al., 2019; Organista et al., 2019). The sections that follow review evidence from COVID-19 pandemic research and then situate Latino day laborers within this normative context.

## Social norms and the COVID-19 pandemic

Research conducted across multiple countries has demonstrated that norms can influence pandemic-related behaviors, including social distancing, mask-wearing, and vaccination (English et al., 2023; Lin et al., 2025). Strong perceived norms have been consistently associated with greater compliance and with mental health–related outcomes, as individuals who perceive widespread engagement in protective behaviors report lower stress and better emotional coping, reflecting reduced uncertainty and stronger collective efficacy during public health crises. When descriptive and injunctive norms are aligned, together they exert a stronger influence because individuals perceive a behavior as being common and understand it as socially approved (Cialdini et al., 1991). This influence is further amplified when individuals strongly identify with the reference group and are motivated to meet its expectations (Lapinski & Rimal, 2005).

In contrast, when motivation to comply is weak, normative messages lose persuasive power, even when descriptive and injunctive cues are present. Ryoo and Kim (2023) have reported that injunctive norms increase masking and distancing in a U.S. sample but only among individuals who believe that maintaining group harmony is important. Together, these studies indicate that to exert behavioral influence, norms require the salience of a reference group and sufficient motivation to comply with this perceived pressure. This evidence highlights the importance of identifying the social groups to which individuals are motivated to respond, as the influence of norms may depend on their specific influence.

Despite the evidence that social norms influence behavior depending on the perceived influence of the referent, most COVID-19 studies have focused on the overall effects of perceived norms, collapsing reference group influence into a single measure of “what people around you do” (Martínez et al., 2021). This leaves unanswered which group exerts influence in deciding whether to comply with a health-related norm. This omission may be critical because reference groups represent different components of an individual’s social network, and expectations from each can exert unequal influence on health-promoting behaviors (Lapinski & Rimal, 2005). Family and peers may exert stronger influence on everyday health behaviors, and expectations within close social networks can shape decisions about vaccination, as individuals describe familial and societal pressure or expectations surrounding COVID-19 vaccine uptake (Lin et al., 2022). Indeed, the referent-specificity of normative influence on COVID-19 vaccination has been demonstrated empirically: perceived vaccination norms from close referents such as friends and family show the strongest associations with vaccination intentions, and this relationship attenuates systematically as the reference group expands to larger, more distal aggregates such as city residents or national populations (Rabb et al., 2022; Zhuang et al., 2024). However, these relationships assume both emotional and physical proximity, even when in the case of Latino day laborers some of the close referents may reisde in other countries. Identifying and comparing referent influence may be essential for understanding when and how norms operate on mitigating and preventive behaviors during a pandemic.

## Latino day laborers in the pandemic

Latino day laborers are mostly foreign-born men who take informal, cash-paid jobs in construction, landscaping, or moving services. During the pandemic, they faced an exceptionally high risk of COVID-19 exposure, as they lacked reliable health information, adequate COVID-19 protective equipment at home and at work, and appropriate access to necessary healthcare (Page et al., 2020). Beyond COVID-19, Latino immigrants also faced broader structural barriers, including a lack of legal protection, economic precarity, and language barriers, which exacerbated health disparities and their ability to take practical preventive actions (Lopez et al., 2021; Viruell-Fuentes et al., 2012). Research on vaccination hesitancy further highlights their vulnerability: Latino communities, including jornaleros, were less likely to receive vaccines early in the rollout, often due to limited access to trusted health information, inconsistent outreach in their preferred language, and high reliance on informal social networks, which often promoted misinformation or disinformation (Abad et al., 2023; Garcia et al., 2021).

The specific referent groups examined in this study, including family, friends, neighbors, coworkers, and fellow day laborers at the corner, were selected based on their theoretical relevance and prominence in prior research on Latino day laborers’ social networks (Negi et al., 2013). Each group represents a distinct relational context through which normative influence may operate. Family is central to Latino cultural life and embodies obligations of emotional attachment, loyalty, and reciprocal responsibility that characterize familismo (Katiria Perez & Cruess, 2014; Sabogal et al., 1987). Neighbors and some friends share residential proximity and cultural familiarity, and may serve as informal conduits for health information and social support. Coworkers and fellow day laborers at the corner share occupational and physical context, economic vulnerability, and daily exposure risk, making them contextually salient referents for health decisions embedded in informal work environments. Because each referent group differs in the nature and intensity of its relationship with the worker, these groups are likely to exert normative influence through distinct pathways. Comparing their relative effects, rather than collapsing referents into a single aggregate measure, is therefore essential for understanding how normative processes operate within this population.

The experience of Latino day laborers during the pandemic illustrates not only the practical but also the theoretical significance of understanding social norms, as these workers operated under compounded uncertainty—economic, health, and social—which heightened their lack of access to information and resources, and their need for cultural guidance based on informal social cues to guide their health decisions. Their behaviors provide a sharper test of social norm theory, because in the absence of consistent or trusted official public health guidance, day laborers may have relied on their reference groups to determine how to protect themselves during the pandemic. For example, observing other workers continue to seek employment may have encouraged risk-taking, increasing the probability of COVID infection. By contrast, experiencing COVID symptoms may have increased their search for what most day laborers do to ameliorate them (a descriptive norm) and what good practices are expected of them while sick in order to maintain income and group belonging (an injunctive norm) (Cialdini et al., 1991). Behavioral cues provided by the social network may have carried greater influence than public health recommendations, particularly when institutional messages felt culturally distant or misaligned with Latino day laborers’ lived experience. This dynamic is consistent with findings that norms exert the strongest influence when they are grounded in referent groups deemed socially meaningful (Foust & Taber, 2024).

## Summary

Latino immigrant day laborers, or jornaleros, represent a population whose social norms remain largely unexplored, even when norms are likely to influence their self-protective health behaviors, including those associated with the prevention or mitigation of COVID-19, including vaccination. This may be an important oversight, as their allocentric cultural orientation suggests they are likely to be susceptible to pressure to comply with others' expectations (social conformity), and that this influence may vary by the type of norm and social referent.

Taken together, prior research provides evidence that social norms have shaped pandemic-related protective behaviors, but their influence may have varied by the type of norm and across different reference groups. Methodological work comparing alternative social norm models suggests that different conclusions may emerge when norms are modeled by norm type, referent group, or specific norm-by-referent combinations (Lac & Donaldson, 2018). What remains unclear is which reference groups were most influential among Latino day laborers and which types of norms (descriptive, injunctive, or normative beliefs) influenced their COVID-19 vaccination behavior. Norms may have been critically important for vaccination, as their influence could have countered the influence of misinformation and vaccination hesitancy. The present study addresses these gaps by comparing three norm types across multiple referent groups simultaneously, and by examining how their relative influence shifted across two phases of the pandemic. A key open question is whether family norms remain dominant even when family members are geographically distant, as is common among day laborers who often maintain transnational ties, and whether this influence extends beyond that of physically proximate referents such as coworkers and fellow workers at the corner. Answering these questions advances theoretical understanding of when and why certain social ties carry more normative weight, and offers practical insight for designing norm-based interventions in populations navigating compounded uncertainty.

The present study examines the relative influence of injunctive and descriptive social norms on COVID-19 vaccination compliance among Latino day laborers and their reference groups, including family, friends, neighbors, coworkers, and fellow day laborers. Specifically, we addressed three research questions: (1) Which individual social norm items were associated with COVID-19 vaccine compliance? (2) Were overall norm-type scores, including descriptive norms, injunctive norms, and normative beliefs, associated with COVID-19 vaccine compliance? (3) Which referent-specific norm scores were associated with COVID-19 vaccine compliance? By distinguishing between overall norm types and referent-specific influences, this study clarifies how normative processes operate among workers living in precarious conditions and identifies which of their social referents exert the greatest influence on their preventive health behavior.

## Methods

### Study design

Data were collected as part of a three-phase, cluster-randomized trial called Vale+Tu Salud, which tested an intervention program to promote preventive behaviors and healthy practices to reduce the impact of the COVID-19 pandemic on Latino Day Laborers (LDLs) in Houston, Texas. For this report, we used data from Phase 1 (2021) and Phase 2 (2023) of the study. The present analysis used baseline(pre-test) data collected before participants were exposed to the intervention in each phase. No prior peer-reviewed publications have reported analyses from the Phase 1 and Phase 2 datasets used in the present manuscript.

### Participants and recruitment

Participants eligible for Phase 1 and Phase 2 of the study had to be 18 years of age or older; self-identify as Latino; and have a history of seeking work at a corner. We define “corner” as public locations where LDLs gather and wait to be hired by contractors. For both data collection periods, a cluster randomized design was employed, whereby corners were randomly selected from a comprehensive list of previously observed corner locations in Houston, Texas. Phase 1 data were collected from March to June 2021 and included 199 participants. Phase 2 data were collected from November 2023 to February 2024 and included 359 participants. Six participants took part in both phases; all other participants were unique to one phase. Because analyses were conducted separately by phase, this limited overlap did not create duplicate observations within any phase-specific model.

The sample size for each phase was determined by the design and recruitment targets of the parent Vale+Tu Salud cluster-randomized trial, in which hiring corners served as the unit of randomization In Phase 1, corners were allocated to two study conditions, with a target of approximately 100 participants per condition, or 200 participants total. In Phase 2, corners were allocated to three study conditions, with a target of approximately 120 participants per condition, or 360 participants total. Parent-trial sample-size planning was based on the cluster-randomized design and power considerations using a two-sided α = 0.05 and target power of at least 80% to detect meaningful differences in mitigation outcomes. Recruitment occurred during the planned data-collection period for each phase and stopped when the recruitment target was reached or the data-collection period ended. No stopping rule based on vaccination outcomes, statistical significance, or interim analyses was used.

### Data collection procedures

Both Phase 1 and Phase 2 used the same data collection procedures. Survey data were collected by a trained Latino outreach team whose members were familiar with Latino day laborers and fluent in Spanish. All interviews were administered in Spanish. English-language ability was not assessed as a separate survey measure because prior survey experience with Latino day laborers in Houston indicated very limited written and verbal English proficiency in this population (Ibekwe et al., 2022). Team members approached potential participants at the selected recruitment sites to explain the purpose of the study. Those who expressed interest were then screened for eligibility. If eligible, individuals provided informed consent before proceeding with the study interviews. Interviewers used a computer tablet to collect survey data programmed in Qualtrics.

### Measures

#### Demographic variables

Demographic measures included participant’s age, time living in the U.S. (in years), time looking for work on the corner (in years), and education years.

#### Norms variables

The construction of social norm measures was guided by the theoretical definitions provided by Cialdini, including injunctive norms and descriptive norms (Cialdini et al., 1991; Lapinski & Rimal, 2005). To operationalize these constructs for Latino day laborers (LDLs), we developed items that assessed types of norms in relation to key reference groups identified in our previous research and in similar studies (Negi et al., 2013) The measurement of normative beliefs was based on basic definitions provided by Ajzen and Fishbein (1972).

***Injunctive norms.*** Participants’ injunctive norm was assessed using Likert-type items and measured the expectations of key social reference groups, including family, friends, coworkers and church members, although the referents measured in each survey changed as indicated in Table 1. Participants responded to the question: “When you think of the following people, how much do you think they want/expect you to get the Coronavirus (COVID-19) vaccine?” Response options were on a 5-point scale: 1 (Not at All), 2 (A little), 3 (More or less), 4 (Somewhat), and 5 (A Lot) (Phase 1 Cronbach’s α= 0.65; Phase 2 Cronbach’s α = 0.66).

**Table 1:** Social norm measures across referents and study phases

Referent	Phase 1			Phase 2		
	Normative beliefs	Injunctive norm	Descriptive norm	Normative beliefs	Injunctive norm	Descriptive norm
Family	X	X (Family and friends)	X	X	X (Family and friends)	X
Friends			X	X		X
Neighbors				X		X
coworker						
Other jornaleros in Houston	X					
Other co-workers that are not jornaleros	X	X			X	
jornaleros looking for work at your corner			X	X		X
Employers or contractors who hire at the corner	X					
People who attend your church	X		X	X		X

Note. Blank cells indicate that the referent was not assessed for that norm type in the corresponding phase

*Descriptive norms.* Participants’ descriptive norm was assessed using Likert-type items to measure the perceived prevalence of COVID vaccination for each of the above mentioned reference groups. Participants responded to the question: “Can you tell me to the best of your knowledge how many of the following individuals have gotten the Coronavirus (COVID-19) vaccine. The response options were on a 4-point scale: 1 (None of them), 2 (Some of them), 3 (Most of them), and 4 (All of them) (Phase 1 Cronbach’s α= 0.79; Phase 2 Cronbach’s α = 0.80).

*Normative beliefs.* Participants’ normative beliefs were assessed using Likert-type items to measure the extent to which specific referent groups were perceived to care about the participant being vaccinated against COVID-19. Participants responded to the question: “How much do you think that the following individuals will care about you being vaccinated against Coronavirus (COVID-19)?” response options were on a 5-point scale: 1 (Not at All), 2 (A little), 3 (More or less), 4 (Somewhat), and 5 (A Lot) (Phase 1 Cronbach’s α= 0.90; Phase 2 Cronbach’s α = 0.83).

*Change in referents.* It must be noted that the reference groups and types of norms measured differed for the two surveys (Table 1). In phase 1, the survey prioritized a broader set of referents across norm types, and the reference groups were not assessed uniformly across all norm types. Normative beliefs were measured for family, other jornaleros in Houston, other co-workers that are not jornaleros, employers or contractors who hire at the corner, people who attend the church. Descriptive norms were measured for family, friends, jornaleros looking for work at the corner, and people who attend church. And injunctive norms were measured for two distinct reference groups: (1) ‘family and friends’ as a single referent group, and (2) coworkers (Table 1). After reviewing Phase 1 data and field experience, the survey team revised the Phase 2 instrument to increase measurement precision and improve alignment between descriptive norm and normative belief referents. This revision allowed the same referent groups to be assessed across these two norm types and supported more consistent referent-specific comparisons. Specifically, the Phase 2 survey improved alignment between descriptive norm and normative belief referents by using the same set of referent groups for both measures: family, friends, neighbors, jornaleros looking for work at the corner, and people who attend church. Injunctive norms were measured for two distinct reference groups: (1) 'family and friends' as a single referent group, and (2) coworkers (Table 1). Because referents were more consistently aligned across norm measures in Phase 2, referent-specific norm scores were calculated only for Phase 2.

#### COVID-19 vaccination compliance

The primary outcome for this study was vaccine compliance, which was defined as having received at least one dose of the COVID-19 vaccine within the 12 months prior to the interview. This binary outcome was constructed using a two-part assessment. First, participants were asked, “Have you received at least one dose of the Coronavirus (COVID-19) vaccine?” (1=No, 2=Yes). If a participant responded "Yes," they were then asked to provide their vaccination date. The vaccine compliance variable was then coded "1" (Yes) only if the participant's self-reported date was within the 12 months prior to their interview. Participants were coded as "0" (No) if they either responded "No" to the initial question or if they responded "Yes" but provided a date that was more than 12 months prior.

### Statistical analysis

The purpose of the statistical analysis was to determine the influence of social norm on COVID vaccine compliance behavior by addressing three primary research questions: (1) to examine whether different individual referents exert a differential influence on vaccination; (2) to assess the cumulative effect of norm across reference groups; (3) to evaluate the differential influence of various social reference groups.

Descriptive statistics were calculated to summarize the sample characteristics. Frequencies and percentages were used for categorical variables, while means and standard deviations (SD) were used for continuous variables. Spearman correlations among social norm measures were calculated separately by phase to assess empirical overlap among norm constructs.

To determine the influence of social norms on COVID-19 vaccination compliance status, three distinct multivariable logistic regression models were performed to investigate the influences of norms on the COVID-19 vaccination compliance. Each model was adjusted for the following demographic covariates: age, years of education, time in the United States (in years), and time looking for work on the corner (in years). Because all social norm and vaccination compliance variables analyzed here were measured at baseline before intervention exposure, intervention condition was not included as a covariate. Results are presented as Odds Ratios (OR) with their corresponding 95% Confidence Intervals (CI).

**Research question 1: differential influence of individual norm items:** To examine whether individual norm items were differentially associated with vaccination compliance, a multivariable logistic regression model was developed using a purposeful selection method (Bursac et al., 2008; Jr et al., 2013). The process involved the following steps: (1) a univariate logistic regression analysis was performed for each individual norm item against the vaccine compliance outcome. (2) any items with p-value <0.10 in the univariate analysis were considered for the multivariable model. All candidate variables were entered into an initial multivariable model. A purposeful selection approach was used, in which the least significant non-confounding variable was considered for removal at each step. Variables with p > .05 were removed only if their removal did not change the coefficients of the remaining significant predictors by more than 20% (Bursac et al., 2008). As a sensitivity analysis, we also estimated models in which all theoretically relevant individual norm items were entered simultaneously with the same demographic covariates.

**Research question 2: cumulative effect of norm types.** To assess the cumulative effect of norm across reference groups, three overall norm scores were calculated by averaging all valid responses from the different referent items within the same norm type scale. This resulted in three distinct scores: a descriptive norm score, an injunctive norm score, and a normative beliefs score. A second multivariable logistic regression model was then conducted including these three overall norm scores simultaneously as independent variables.

**Research Question 3: differential influence of social referents.** To evaluate the differential influence of various social reference groups, referent-specific norm scores, defined as the mean of all norm types for a single referent, were created. This analysis was only feasible for Phase 2, as this was the only phase where referents were assessed separately and consistently. Because only the descriptive norm and normative belief items aligned across the same set of referents (Table 1), the final referent-specific scores were calculated as the mean of just those two items for a given referent (e.g., Family Norm = mean of family descriptive norm and family normative belief). This process generated five referent-specific scores: family norm, friends norm, neighbors norm, jornaleros norm, and church norm. A third multivariable logistic regression model was conducted with these referent-specific scores entered simultaneously as independent variables. The church norm was excluded from this analysis due to a high prevalence of non-applicability (34.3% of participants reported no attendance), which would have substantially reduced the analytic sample size.

All statistical analyses were performed using STATA (Version 15.SE, StataCorp, College Station, TX). The alpha level for determining statistical significance was set at *p* < 0.05 for all tests. The Social Norm Predictor Variables construction detail is shown in Table 2.

**Table 2:** Construction of social norm predictor variables

Set	Variable name(s)	Phase	Component items included in mean score
Overall score	Descriptive Norm	Phase 1	DS-Family, DS-Friends, DS-Jornaleros, DS-Church
		Phase 2	DS-Family, DS-Friends, DS-Neighbors, DS-Jornaleros
	Injunctive Norm	Phase 1	IN-(Family/Friends), IN-Coworker
		Phase 2	IN-(Family/Friends), IN-Coworker
	Normative Beliefs	Phase 1	NB-Family, NB-Other Jornaleros, NB-Other Coworkers, NB-Employers, NB-Church
		Phase 2	NB-Family, NB-Friends, NB-Neighbors, NB-Jornaleros
Referent-specific norm scores	Family Norm	Phase 2	DS-Family, NB-Family
	Friends Norm	Phase 2	DS-Friends, NB-Friends
	Neighbors Norm	Phase 2	DS-Neighbors, NB-Neighbors
	Jornaleros Norm	Phase 2	DS-Jornaleros, NB-Jornaleros

## Results

The demographic characteristics of the 199 participants from Phase 1 and 359 participants from Phase 2 are detailed in Table 1. In Phase 1, the average participant was approximately 46.7 years old (SD = 12.2), had lived in the US for about 15.0 years (SD = 13.3), and had 8.1 years of education. The Phase 2 sample was, on average, slightly younger (mean age = 42.5, SD = 13.4) and had lived in the US for a shorter time (mean = 10.2 years, SD = 11.4). Regarding baseline (pre-test) vaccination status, vaccine compliance was 50.6% in Phase 1 and dropped to 25.6% in Phase 2.

Regarding the social norm measures (Table 3) a comparison to their respective scale midpoints reveals a notable pattern. In Phase 1, injunctive norms were, on average, endorsed above their scale's midpoint (mean = 3.20, on a 1–5 scale). In contrast, descriptive norms were endorsed below their midpoint (mean = 2.35, on a 1–4 scale). In Phase 2, both injunctive norms (mean = 2.49, on a 1–5 scale) and descriptive norms (mean = 2.42, on a 1–4 scale) were, on average, endorsed below their respective midpoints. Among the referent-specific norms (measured only in Phase 2), family norms (mean = 3.22, SD = 1.07) were the most highly endorsed, while neighbor norms (mean = 2.27, SD = 0.87) were the least. Spearman correlations among social norm variables were examined separately by phase and are presented in Supplementary Tables S2a and S2b to describe empirical overlap among the norm measures.

**Table 3:** Descriptive statistics of demographic characteristics and social norms

Factor	Phase 1 (N=199)		Phase 2 (N=359)	
	N	Value†	N	Value†
Age	195	46.70 (12.21)	353	42.47 (13.41)
Time in US	194	14.98 (13.32)	358	10.24 (11.37)
Time on the corner	189	5.13 (6.52)	359	3.77 (5.76)
Education years	198	8.11 (4.59)	359	7.60 (4.24)
Descriptive norm	198	2.35 (0.75)	357	2.42 (0.69)
Injunctive norm	199	3.20 (1.11)	359	2.49 (0.99)
Normative beliefs	199	2.99 (1.13)	344	2.88 (1.15)
Family norm			339	3.22 (1.07)
Friends norm			320	2.45 (0.91)
Neighbors norm			271	2.27 (0.87)
Jornaleros norm			282	2.48 (0.94)
Church norm			236	2.68 (1.06)
Vaccine compliance ^a^				
Yes	86	50.59%	91	25.60%
No	84	49.41%	264	74.40%

^†^Values are presented as mean (SD) for continuous variables and n (%) for categorical variables

^a^Vaccine Compliance represent baseline (pre-test) results for both Phase 1 and Phase 2 prior to intervention

### Phase 1 findings

**Fig. 1:**
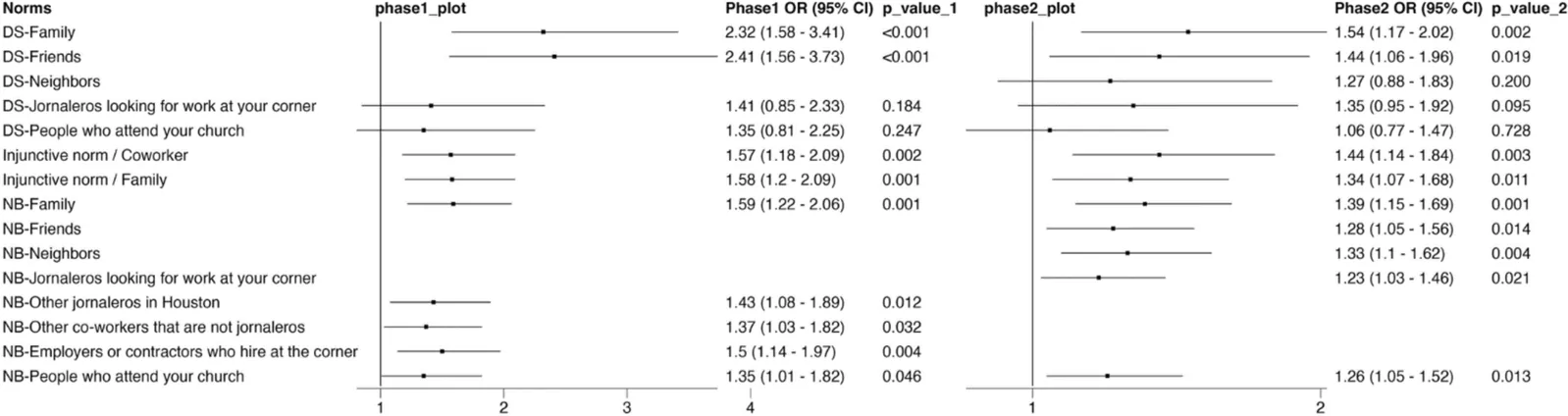
Adjusted associations between individual social norm items and COVID-19 vaccine compliance among Latino day laborers, by study phase. Note. *Odds ratios (ORs) and 95% confidence intervals (CIs) were estimated from separate logistic regression models adjusted for age, years of education, time in the United States, and time seeking work at the corner. *DS = descriptive norm; IN = injunctive norm; NB = normative belief. *A dash (—) indicates that the item was not assessed in that study phase. The vertical reference line indicates OR = 1.0

***Differential influence of individual norm items.*** Figure 1 presents the adjusted associations between each individual social norm item and COVID-19 vaccine compliance in Phase 1. Several perceived social norms from close referents, specifically family, friends, and coworkers, were significantly associated with higher odds of vaccination compliance. Descriptive norms from both family (OR = 2.32, 95% CI [1.58, 3.41], *p* < 0.001) and friends (OR = 2.41, 95% CI [1.56, 3.73], *p* < 0.001) were significantly associated with vaccine compliance. The influence of family was pervasive, showing a significant association across all three norm types: descriptive, injunctive, and normative beliefs (p<0.001). Other significant associations included injunctive norms from coworkers, and normative beliefs from several work-related referents, such as coworkers and employers (Fig. 1).

After conducting model selection, Table 4 shows the results from the final multivariable logistic regression model examining the association between social norms and vaccine compliance, after adjusting for demographic characteristics. Descriptive norms related to family (DS-Family) were significantly associated with higher odds of vaccine compliance (OR = 1.976, 95% CI [1.297–3.011], *p* = 0.002). Additionally, normative beliefs related to family (NB-Family) showed a positive trend but were not statistically significant (OR = 1.310, 95% CI [0.975–1.759], *p* = 0.073). A sensitivity analysis including all theoretically relevant individual norm items are presented in Supplementary Table S1a.

**Table 4:** The multivariable logistic regression between social norms and vaccine compliance, adjusting for demographic characteristics

Variable	Odds ratio	p-value	LL	UL
Phase 1				
*Model 1a: differential influence of individual norm items*				
DS-family	1.976	**0.002**	1.297	3.011
Normative beliefs-family	1.31	0.073	0.975	1.759
*Model 2a: cumulative effect of norm types*				
Descriptive norm	2.443	**0.012**	1.213	4.918
injunctive norm	1.507	0.058	0.987	2.300
Normative beliefs	1.052	0.832	0.66	1.677
Phase 2				
*Model 1b: differential influence of individual norm items*				
DS-family	1.312	0.069	0.979	1.757
NB-family	1.303	**0.010**	1.066	1.593
*Model 2b: cumulative effect of norm types*				
Descriptive norm	1.138	0.567	0.731	1.772
Injunctive norm	1.353	0.062	0.985	1.857
Normative beliefs	1.347	**0.047**	1.004	1.807
*Model 3: differential influence of social referents*				
Family	1.677	**0.009**	1.141	2.466
Friends	1.034	0.901	0.611	1.75
Neighbors	1.041	0.887	0.597	1.817
Jornaleros	1.000	0.999	0.617	1.618

Note. Values are adjusted odds ratios (aORs) and 95% confidence intervals (CIs). Bold p-values indicate statistical significance at p < 0.05.

All models were adjusted for age, years of education, time living in the United States, and time seeking work at the corner

Model 1 examined individual social norm items. Model 2 examined overall norm-type scores, including descriptive norms, injunctive norms, and normative beliefs. Model 3 examined referent-specific norm scores and was conducted only in Phase 2 because referents were more consistently aligned across norm measures in that phase

***Cumulative effect of norm types.*** Table 4 presents the results of logistic regression analyses examining the association between different types of social norms and vaccine compliance, adjusting for the demographic characteristics. Descriptive norm was significantly associated with vaccine compliance (aOR = 2.443, 95% CI [1.213, 4.918], *p* = 0.012).

### Phase 2 findings

***Differential influence of individual norm items.*** Figure 1 also presents the adjusted associations between each individual social norm item and COVID-19 vaccine compliance in Phase 2. In this phase, the pattern of associations was broader but generally weaker than in Phase 1. The strongest association was observed for descriptive norms related to family (OR=1.538, 95% CI [1.173–2.017], p=0.002). Similarly, descriptive norms from friends were also significantly associated with vaccination compliance. Injunctive norms showed a similar pattern, with those from coworkers and the combined "family and friends" group also being significant. Furthermore, normative beliefs were significant across a broad range of referents, including family, friends, neighbors, and jornaleros at the corner (Fig. 1).

In phase 2, while descriptive norms still showed a positive trend, normative beliefs emerged as the primary significant factor. Normative beliefs related to family (NB-Family) were a significant predictor (OR = 1.303, 95% CI [1.066–1.593], *p* = 0.010). Descriptive norms related to family (DS-Family) indicated a marginally significant association (OR = 1.312, 95% CI [0.979–1.757], *p* = 0.069) (Table 4). Sensitivity analyses entering all theoretically relevant individual norm items simultaneously with the same demographic covariates are presented in Supplementary Table S1b.

***Cumulative effect of norm types.*** The analysis of overall norm scores revealed that normative beliefs were significantly associated with higher odds of vaccine compliance. (aOR = 1.347, 95% CI [1.004, 1.807], *p* = 0.047). Injunctive norms showed a trend toward significance with a similar odds ratio (aOR = 1.353, 95% CI [0.985, 1.857], *p* = 0.062), while descriptive norms were not significantly associated with vaccination (Table 4).

***Differential influence of social referents.*** Finally, to assess whether normative influence varied across referents, model 3 examined the referent-specific norm scores (computed for family, friends, neighbors, jornaleros). Table 4 shows a strong, statistically significant association found for family norms (aOR = 1.677, 95% CI [1.141, 2.466], *p* = 0.009). In contrast, norms from friends, neighbors, and jornaleros were not significantly associated with vaccine compliance (Table 4).

In summary, our findings suggest a phase-specific and differential pattern of associations between social norms and vaccination compliance. In Phase 1, fewer norm items emerged as significant, but the significant associations were clearer and generally larger, particularly descriptive norms from family and friends. In Phase 2, a broader set of norm items was associated with vaccination compliance, but the magnitude of associations was generally smaller and no single predictor stood out as strongly as the leading Phase 1 predictors. Across both the individual-item and composite norm models, the most influential norms shifted over time from family descriptive norms (what family does) in Phase 1 to family normative beliefs (how much family cares about vaccination) in Phase 2. Furthermore, family-related norms were the most consistent normative correlates of vaccination compliance among Latino day laborers. Work-related referents also showed some associations, particularly in Phase 1, but these associations were less consistent than family-related norms.

## Discussion

This study advances understanding of how social norms shape COVID-19 vaccination behavior among Latino day laborers by disentangling norm type (descriptive, injunctive, and normative beliefs) from reference group, and by examining how these influences shifted over time. Consistent with social norms theory (Lapinski & Rimal, 2005), our findings demonstrate that normative influence was neither uniform nor static. Instead, vaccination behavior was shaped by a dynamic interplay between what individuals perceived others were doing, what they believed others expected of them, and whose expectations mattered most.

### Differential influence of norm types over time

Across the two study phases, the dominant form of normative influence shifted from descriptive norms to normative beliefs, indicating that the processes motivating vaccination evolved as the pandemic progressed. In Phase 1, vaccination compliance was most strongly associated with descriptive norms, particularly perceptions that family members were getting vaccinated. This pattern is consistent with early pandemic conditions characterized by uncertainty and limited access to clear institutional guidance, when observing others’ behavior served as a practical heuristic for action (Lapinski & Rimal, 2005). This interpretation is reinforced by recent longitudinal evidence tracking norm perceptions across two years of the pandemic, which found that descriptive norms most strongly drove protective behavior during periods of heightened uncertainty, with their influence diminishing as the pandemic progressed (Heiman et al., 2023). For day laborers operating in informal work environments and facing heightened exposure risk, perceptions of what close others were doing may have provided critical cues for navigating unfamiliar health decisions. This influence is particularly important, as it did not involve frequent or face-to-face contact, since the families of day laborers were likely living outside the United States.

By Phase 2, normative beliefs emerged as the primary predictor of vaccination compliance. This transition suggests that as vaccines became more widely available and public discourse around vaccination intensified, decisions shifted from observing others’ actions to being influenced by perceived social expectations and accountability. Normative beliefs reflect not only perceived approval but also motivation to comply with the expectations of important others (Ajzen, 1991; Lapinski & Rimal, 2005). Their increasing salience may indicate a maturation of normative processes, in which vaccination became framed not simply as a common behavior but as a socially meaningful and morally weighted decision.

This phase-specific pattern also helps explain why Phase 1 showed clearer and generally larger individual-item associations, whereas Phase 2 showed a broader but weaker pattern of associations. Earlier in the pandemic, vaccination was relatively new, and participants may have relied on a smaller number of highly salient social cues, particularly descriptive norms from close referents such as family and friends. Later, as vaccination became more familiar and public discussion became more established, remaining noncompliance may have reflected a more complex combination of structural barriers, accumulated mistrust, entrenched hesitancy, or competing information sources. Under these conditions, individual norm items may have had less ability to clearly distinguish compliant from non-compliant participants, producing a broader but weaker pattern of associations in Phase 2. Thus, the observed pattern suggests a possible movement from reliance on behavioral cues to reliance on perceived social expectations over time. However, this interpretation should be considered cautiously because the two phases were analyzed separately and the norm measures were not fully parallel across phases.

### Central role of family as a normative referent

Across both phases and analytic approaches, family consistently emerged as the most influential reference group, surpassing friends, coworkers, neighbors, fellow day laborers, and faith-based networks. Family norms were the only referent that remained significantly associated with vaccination compliance in referent-specific models, underscoring the centrality of kinship ties in shaping health behavior among Latino day laborers.

This finding aligns with prior work identifying familismo as a multidimensional cultural value in Latino communities. Early conceptual and empirical work characterizes familismo as emphasizing strong emotional attachment, loyalty, and reciprocal obligation within the family unit, shaping how individuals prioritize family needs in everyday decision-making (Sabogal et al., 1987). Subsequent reviews have shown that these family-centered values are consistently associated with health-related behaviors and outcomes among Latino populations, particularly when decisions involve perceived responsibility to protect or support family members (Katiria Perez & Cruess, 2014). More recent meta-analytic evidence further suggests that familism values are associated with behavioral adjustment and decision-making through perceived obligations and concern for family well-being, rather than solely through generalized social influence (Cahill et al., 2021).

Within this framework, our findings suggest that family influence on vaccination behavior may operate through distinct normative pathways over time—initially through perceptions of family members’ behavior and later through perceived expectations and concern for family approval. This pattern highlights why interventions that treat social norms as undifferentiated or interchangeable across reference groups may fail to capture the culturally specific processes through which normative influence operates in Latino populations.

### Limited influence of other social referents

Despite theoretical expectations that peers, coworkers, and fellow day laborers might exert substantial normative influence, particularly given shared occupational risks and frequent daily interactions, these groups did not independently predict vaccination compliance in multivariable models. From a social norms perspective, coworkers are often expected to influence health behaviors because they shape context-specific norms related to workplace safety, risk tolerance, and acceptable protective practices, especially in settings where exposure risk is shared and formal institutional guidance is limited. However, while univariate analyses suggested that norms from these referents were associated with vaccination behavior, their effects attenuated once family norms were considered simultaneously. This pattern suggests that, for Latino day laborers, vaccination decisions may be governed less by situational workplace norms and more by enduring social obligations and expectations rooted in family relationships, which carry greater emotional salience and long-term accountability. This interpretation is consistent with recent evidence that expectations from close social ties exert a stronger influence on COVID-19 vaccination decisions than broader societal norms (T. Lin et al., 2025).

It is also important to acknowledge that differences in measurement structure across norm types and study phases, particularly the inability to construct fully parallel referent-specific norm scores in Phase 1, may have constrained the detection of some non-family normative effects. However, the consistency of family effects across individual-item, composite, and referent-specific models suggests that the central role of family norms represents a robust pattern rather than a measurement artifact.

This pattern suggests that normative influence among day laborers may be hierarchical rather than additive, with family norms taking precedence over other social cues. It also aligns with prior research indicating that trust in informal social networks does not uniformly translate into health-related outcomes among Latino day laborers (Catindig et al., 2024). Taken together, these findings caution against assuming that proximity or frequency of interaction alone determines normative power; instead, perceived legitimacy, emotional salience, and long-term relational obligations appear to be more decisive.

These findings can be further understood through the lens of social identity and social categorization theory, which propose that normative influence depends not only on proximity or frequency of contact but on the perceived psychological significance of the reference group (Tajfel & Turner, 1979). From a social categorization perspective, referents exert stronger normative pull when they are perceived as meaningful ingroup members whose approval or disapproval carries identity-relevant consequences (Turner et al., 1987). This proposition has empirical support: within the theory of planned behavior, group norms predict intentions most strongly among individuals who identify closely with the referent group (White et al., 2009). Pandemic-specific evidence follows the same pattern, as descriptive norms attributed to a more self-relevant neighborhood group predicted COVID-19 protective behaviors more strongly than norms attributed to the more distal national group in a three-wave longitudinal study (Zhou et al., 2023). For Latino day laborers, family may occupy a position of heightened psychological salience precisely because it represents the primary locus of identity, obligation, and long-term accountability, regardless of geographic distance. These findings also speak to the local dominance effect, which proposes that normative influence is strongest from referents perceived as personally immediate and relevant (Zell & Alicke, 2010). Crucially, our results suggest that “local” in this context operates emotionally and culturally rather than spatially: family exerted stronger normative influence than physically co-present referents such as coworkers or fellow day laborers. This implies that social identity and felt obligation, rather than physical proximity, determine which referents are psychologically “near” for this population.

### Theoretical implications for social norms research

By explicitly distinguishing norm types and reference groups, this study responds to longstanding calls for greater conceptual precision in social norms research, which have emphasized the need to differentiate descriptive, injunctive, and referent-specific norms to better understand when and how normative influence operates (Lapinski & Rimal, 2005; Rimal & Real, 2005). Much of the COVID-19 literature has relied on aggregate measures of perceived norms that obscure whose behavior or expectations are being referenced. Our findings demonstrate that such aggregation risks masking critical variation, particularly in populations whose social networks are structured around culturally specific hierarchies of influence.

Moreover, the observed temporal shift from descriptive norms to normative beliefs provides empirical support for theoretical models that propose the motivation to comply moderates the effect of norms (Rimal & Real, 2005). Normative cues were not uniformly persuasive; instead, their influence depended on whether individuals felt accountable to the reference group and motivated to meet its expectations. This dynamic helps reconcile mixed findings in prior pandemic research and underscores the importance of situating normative influence within broader social and temporal contexts.

### Implications for public health practice

Although this study was not designed to evaluate interventions, the findings have implications for public health communication strategies targeting Latino day laborers and similar populations. Messaging that emphasizes generalized community behavior may be insufficient, particularly as a public health crisis evolves. Strategies that recognize family-centered decision-making and frame preventive behaviors as consistent with family expectations and responsibilities may be more effective than approaches relying solely on peer or workplace norms. Consistent with this, a community-partnered intervention delivering culturally tailored, family-oriented vaccine messaging to low-income, Spanish-speaking immigrant Latino families produced measurable improvements in vaccination outcomes relative to a control condition (Castellon-Lopez et al., 2024).

### Limitations and future directions

Several limitations should be acknowledged. First, the study relied on self-reported vaccination status and perceived norms, which may be subject to recall or social desirability bias. Second, differences in norm measurement across phases constrained direct longitudinal comparisons and required phase-specific analytic strategies. Third, the cross-sectional design of each phase limits causal inference regarding the directionality of normative influence. Because vaccination may have occurred before survey completion, participants’ vaccination status may have shaped their perceptions of others’ behaviors or expectations. Findings should therefore be interpreted as associations rather than evidence of causal influence.

Future research should explore how family norms are communicated and negotiated within households, particularly in transnational or mixed-status families. Longitudinal designs could further clarify how normative influence evolves across different stages of public health emergencies. Additionally, qualitative work may help unpack why certain referents—such as fellow day laborers, who carry less influence than they are theoretically expected to.

## Conclusion

This study examined how subjective norms shaped COVID-19 vaccination behavior among Latino day laborers during a period of substantial uncertainty. The findings demonstrate that both norm type and reference group mattered for vaccination compliance, and that their relative importance varied across the pandemic's phases. In particular, normative influence shifted over time from descriptive norms to normative beliefs, indicating that the processes motivating vaccination evolved as information availability and social expectations changed.

Across all analyses, family emerged as the most consistent and influential reference group, underscoring the central role of family-centered values and expectations in shaping health-related decision making among Latino men. These results indicate that normative influence is not uniform across social ties but is structured by culturally salient relationships that carry enduring social obligations.

More broadly, this study clarifies how normative processes operate under conditions of uncertainty by showing that the influence of social norms depends not only on norm type, but also on whose expectations are perceived as meaningful. By disentangling norm type from reference group, the findings contribute to greater conceptual precision in social norms research while offering insight into how preventive health behaviors are shaped within marginalized worker populations.

## Supplementary Information

Below is the link to the electronic supplementary material.

Supplementary file1 (DOCX 22 KB)

## Data Availability

The datasets analyzed during the current study are not publicly available due to participant confidentiality protections but are available from the corresponding author on reasonable request and with appropriate approvals.
