# Supplementary material for: Beyond social cues: the normative influence of social referents on COVID-19 vaccine compliance among Latino day laborers
Source: J Behav Med. 2026 Sep 16;49(6):1129–42. doi: 10.1007/s10865-026-00709-8 (PMC13748010; doi:10.1007/s10865-026-00709-8)
Supplement: Supplementary file 1 [file 10865_2026_709_MOESM1_ESM.docx]

**Supplementary Table S1.** Comprehensive models including all theoretically relevant individual norm items

**Supplementary Table S1a.** Phase 1 comprehensive model

| **Norms** | **aOR** | **95% CI** | **p value** |
| --- | --- | --- | --- |
| DS-Family | 1.851 | 0.796–4.303 | 0.153 |
| DS-Friends | 1.712 | 0.738–3.967 | 0.210 |
| DS-Neighbors | 1.738 | 0.645–4.683 | 0.274 |
| DS-People who attend your church | 0.353 | 0.117–1.065 | 0.065 |
| IN-Coworker | 1.005 | 0.590–1.712 | 0.984 |
| IN-Family and friends | 1.340 | 0.769–2.333 | 0.301 |
| NB-Family | 1.320 | 0.718–2.428 | 0.371 |
| NB-Friends | 0.842 | 0.428–1.658 | 0.620 |
| NB-Neighbors | 0.820 | 0.389–1.729 | 0.602 |
| NB-Other jornaleros in Houston | 0.785 | 0.428–1.440 | 0.434 |
| NB-People who attend your church | 1.285 | 0.656–2.516 | 0.465 |
| Age | 1.072 | 1.011–1.136 | 0.020 |
| Time in US | 0.976 | 0.928–1.027 | 0.349 |
| Time on corner | 1.021 | 0.911–1.144 | 0.724 |
| Education Years | 1.089 | 0.958–1.239 | 0.193 |

Note. aOR = adjusted odds ratio; CI = confidence interval. Phase 1 models were adjusted for age, time in the United States, time on the corner, and education years. All social norm variables and covariates were entered simultaneously in the model.

**Supplementary Table S1b.** Phase 2 comprehensive model

| **Norms** | **aOR** | **95% CI** | **p value** |
| --- | --- | --- | --- |
| DS-Family | 1.287 | 0.871–1.901 | 0.205 |
| DS-Friends | 1.058 | 0.632–1.770 | 0.831 |
| DS-Neighbors | 0.771 | 0.434–1.369 | 0.374 |
| DS-Jornaleros looking for work at your corner | 1.021 | 0.604–1.727 | 0.938 |
| IN-Family and friends | 1.078 | 0.755–1.539 | 0.678 |
| IN-Coworker | 1.153 | 0.821–1.617 | 0.411 |
| NB-Family | 1.283 | 0.983–1.676 | 0.067 |
| NB-Friends | 0.933 | 0.646–1.349 | 0.714 |
| NB-Neighbors | 1.137 | 0.783–1.652 | 0.500 |
| NB-Jornaleros looking for work at your corner | 0.993 | 0.758–1.301 | 0.961 |
| Age | 1.001 | 0.974–1.030 | 0.917 |
| Time in US | 0.996 | 0.960–1.033 | 0.833 |
| Time on corner | 0.968 | 0.897–1.043 | 0.391 |
| Education Years | 1.055 | 0.971–1.146 | 0.208 |

Note. aOR = adjusted odds ratio; CI = confidence interval. Phase 2 models were adjusted for age, time in the United States, time on the corner, and education years. All individual social norm variables were entered simultaneously in the model.

**Supplementary Table S2.** Spearman Correlation Matrices Among Social Norm Variables by Phase

**Supplementary Table S2a.** Spearman Correlation Matrix Among Phase 1 Social Norm Items

| **Variable** | **1** | **2** | **3** | **4** | **5** | **6** | **7** | **8** | **9** | **10** | **11** | **12** | **13** |
| --- | --- | --- | --- | --- | --- | --- | --- | --- | --- | --- | --- | --- | --- |
| **Item-level social norm variables** |  |  |  |  |  |  |  |  |  |  |  |  |  |
| 1. DS–Family | — |  |  |  |  |  |  |  |  |  |  |  |  |
| 2. DS–Friends | .69* | — |  |  |  |  |  |  |  |  |  |  |  |
| 3. DS–Neighbors | .47* | .45* | — |  |  |  |  |  |  |  |  |  |  |
| 4. DS–Jornaleros | .41* | .53* | .49* | — |  |  |  |  |  |  |  |  |  |
| 5. IN–Family/Friends | .44* | .36* | .22* | .31* | — |  |  |  |  |  |  |  |  |
| 6. IN–Coworker | .33* | .28* | .19* | .12 | .48* | — |  |  |  |  |  |  |  |
| 7. NB–Family | .51* | .43* | .25* | .29* | .60* | .41* | — |  |  |  |  |  |  |
| 8. NB–Friends | .58* | .44* | .37* | .30* | .35* | .45* | .59* | — |  |  |  |  |  |
| 9. NB–Neighbors | .51* | .49* | .33* | .43* | .34* | .38* | .58* | .81* | — |  |  |  |  |
| 10. NB–Jornaleros | .44* | .40* | .27* | .37* | .47* | .35* | .61* | .57* | .64* | — |  |  |  |
| **Overall norm-type scores** |  |  |  |  |  |  |  |  |  |  |  |  |  |
| 11. Descriptive norm score |  |  |  |  |  |  |  |  |  |  | — |  |  |
| 12. Injunctive norm score |  |  |  |  |  |  |  |  |  |  | .42* | — |  |
| 13. Normative belief score |  |  |  |  |  |  |  |  |  |  | .61* | .55* | — |

Note. Values are Spearman’s rank-order correlation coefficients. DS = descriptive norm; IN = injunctive norm; NB = normative belief. p < .05.

**Supplementary Table S2b.** Spearman Correlation Matrix Among Phase 2 Social Norm Items

| **Variable** | **1** | **2** | **3** | **4** | **5** | **6** | **7** | **8** | **9** | **10** | **11** | **12** | **13** | **14** | **15** | **16** | **17** |
| --- | --- | --- | --- | --- | --- | --- | --- | --- | --- | --- | --- | --- | --- | --- | --- | --- | --- |
| **Item-level social norm variables** |  |  |  |  |  |  |  |  |  |  |  |  |  |  |  |  |  |
| 1. DS–Family | — |  |  |  |  |  |  |  |  |  |  |  |  |  |  |  |  |
| 2. DS–Friends | .52* | — |  |  |  |  |  |  |  |  |  |  |  |  |  |  |  |
| 3. DS–Neighbors | .35* | .55* | — |  |  |  |  |  |  |  |  |  |  |  |  |  |  |
| 4. DS–Jornaleros | .35* | .50* | .61* | — |  |  |  |  |  |  |  |  |  |  |  |  |  |
| 5. IN–Family/Friends | .26* | .27* | .21* | .26* | — |  |  |  |  |  |  |  |  |  |  |  |  |
| 6. IN–Coworker | .10 | .17* | .23* | .30* | .47* | — |  |  |  |  |  |  |  |  |  |  |  |
| 7. NB–Family | .35* | .33* | .29* | .31* | .43* | .29* | — |  |  |  |  |  |  |  |  |  |  |
| 8. NB–Friends | .22* | .35* | .37* | .37* | .35* | .44* | .48* | — |  |  |  |  |  |  |  |  |  |
| 9. NB–Neighbors | .16* | .28* | .40* | .36* | .29* | .41* | .40* | .70* | — |  |  |  |  |  |  |  |  |
| 10. NB–Jornaleros | .25* | .30* | .38* | .29* | .29* | .32* | .46* | .53* | .54* | — |  |  |  |  |  |  |  |
| **Overall norm-type scores** |  |  |  |  |  |  |  |  |  |  |  |  |  |  |  |  |  |
| 11. Descriptive norm score |  |  |  |  |  |  |  |  |  |  | — |  |  |  |  |  |  |
| 12. Injunctive norm score |  |  |  |  |  |  |  |  |  |  | .34* | — |  |  |  |  |  |
| 13. Normative belief score |  |  |  |  |  |  |  |  |  |  | .48* | .52* | — |  |  |  |  |
| **Referent-specific norm scores** |  |  |  |  |  |  |  |  |  |  |  |  |  |  |  |  |  |
| 14. Family referent score |  |  |  |  |  |  |  |  |  |  |  |  |  | — |  |  |  |
| 15. Friends referent score |  |  |  |  |  |  |  |  |  |  |  |  |  | .56* | — |  |  |
| 16. Neighbors referent score |  |  |  |  |  |  |  |  |  |  |  |  |  | .43* | .70* | — |  |
| 17. Jornalero referent score |  |  |  |  |  |  |  |  |  |  |  |  |  | .52* | .62* | .67* | — |

Note. Values are Spearman’s rank-order correlation coefficients. DS = descriptive norm; IN = injunctive norm; NB = normative belief. p < .05.
